# Use of Endovascular Coiling to Stop Gastric Ulcer Bleeding in a Patient With Extensive Risk Factors

**DOI:** 10.7759/cureus.16164

**Published:** 2021-07-04

**Authors:** Hassam Ali, Momal Sana, Rahul Pamarthy, Eiman Rahat, Shiza Sarfraz

**Affiliations:** 1 Internal Medicine, East Carolina University, Vidant Medical Center, Greenville, USA; 2 Internal Medicine, Dorevitch Pathology, Heidelberg, AUS; 3 Internal Medicine, Ziauddin University, Karachi, PAK; 4 Anesthesiology, Bahawal Victoria Hospital, Quaid-E-Azam Medical College, Bahawalpur, PAK

**Keywords:** peptic ulcer bleed, tae, transarterial coil embolization, angiographic embolization, pud

## Abstract

Peptic ulcer disease (PUD) can lead to life-threatening bleeding. Endoscopy is a primary intervention used to locate the site of bleeding and maintain hemostasis. When considering multiple risk factors to operative intervention or failed initial endoscopic procedure in patients, the preferred treatment for acute gastrointestinal bleeding remains endovascular coiling to embolize the culprit's vessel. We report a case of a 57-year-old female who presents with melena secondary to gastric ulcer not amenable to endoscopic interventions. Various embolization techniques are available demanding clinicians' attention towards their role in managing ulcer bleeds and their impact on the controlling bleeds.

## Introduction

Peptic ulcer disease (PUD) comprises duodenal and gastric ulcers, where gastric ulcers being more common due to a greater incidence [1]. Incidence depends on various risk factors and co-morbidities, including often overlooked concomitant use of blood thinners and a history of chronic analgesic intake. Management plan algorithm starts from proton pump inhibitors (PPIs), followed by endoscopy, angiography, embolization, or surgery, based on clinical severity. We report a case of a 57-year-old female on chronic anticoagulation for antiphospholipid syndrome who presented with a massive duodenal ulcer refractory to endoscopic measures. The patient was initially resuscitated and treated medically, followed by endoscopic clipping, which failed to provide desired results. Ultimately, she was managed with coil embolization by vascular interventional radiology. We review the effectiveness of the embolization technique using endovascular coiling, managing ulcer bleeds, and its impact on vasculature's anatomical significance [2].

## Case presentation

**Figure 1:**
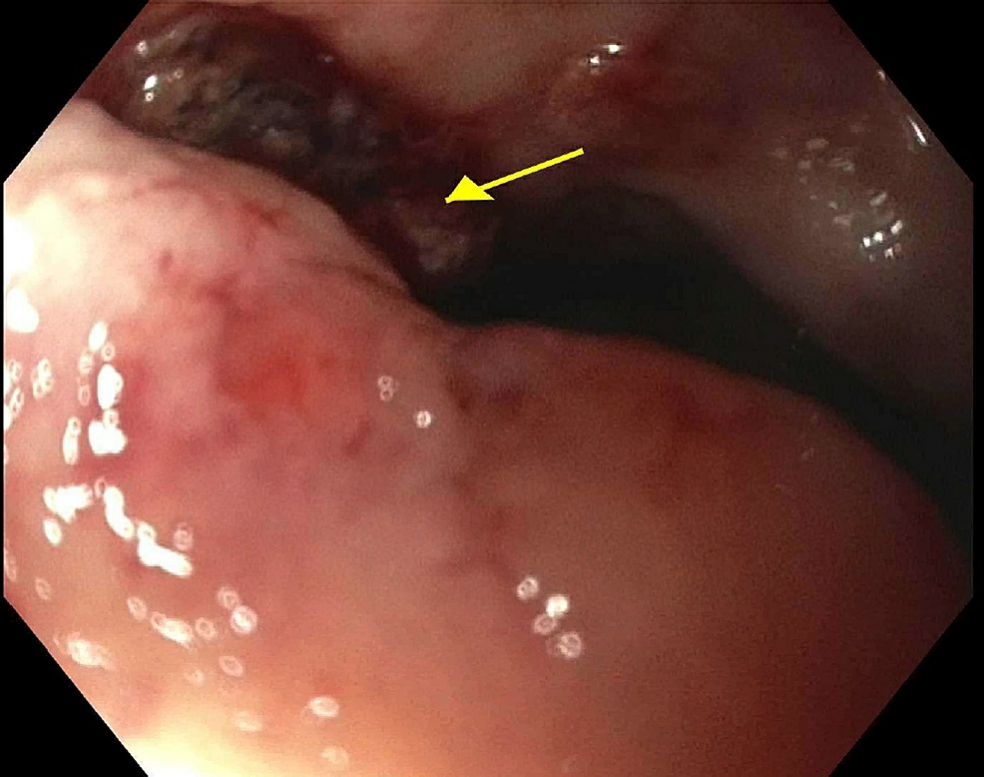
Gastric ulcer within pyloric channel extending into proximal bulb (yellow arrow).

A 57-year-old female with multiple melanic bowel movements for one day presented in the emergency department (ED). Her past medical history was significant for systemic lupus erythematosus (SLE), antiphospholipid syndrome, lacunar infarct, hypertension, and osteoarthritis of both knees’ status post arthroplasty. She denied any abdominal pain, nausea, vomiting, or hematemesis. Due to the subtherapeutic international normalized ratio (INR), low molecular weight heparin (LMWH) was added to her anticoagulation regimen for three days. She was also taking meloxicam chronically for her arthritis and prednisone for SLE. The previous history of gastrointestinal bleed or ulcers was negative. Her basic labs revealed hemoglobin of 5 g/dL, INR 2.5, and platelet count within normal limits. While in the ED, she had ongoing melena but was hemodynamically stable. Her warfarin and LMWH were held, and her INR was reversed with fresh frozen plasma. She also received multiple packed red blood cells for a goal of hemoglobin of 7-9 g/dL. Emergent endoscopy revealed a clot in the duodenum extending into the antrum overlying a large 12 mm gastric ulcer with a visible vessel within the pyloric channel extending into the proximal bulb not amenable to endoscopic clipping (Figure 1).

**Figure 2:**
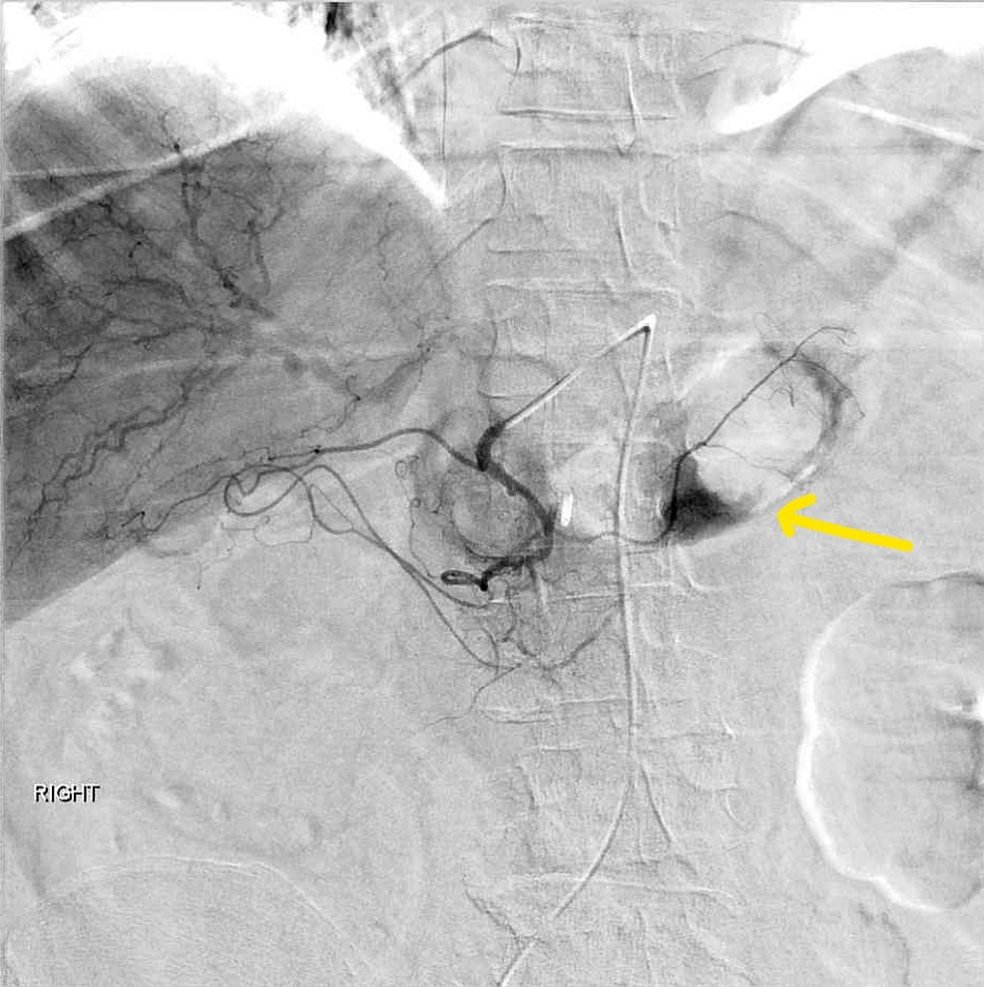
Active extravasation of dye due to the culprit vessel erosion by the ulcer (yellow arrow).

Interventional radiology was consulted, and on the pancreaticoduodenal arteriogram, active extravasation was demonstrated (Figure 2).

Following coil and surgifoam embolization of the gastroduodenal artery (GDA), the post-embolization arteriogram revealed occlusion of this vessel; and the patient underwent successful embolization of the GDA and main pancreaticoduodenal artery supplying the ulcer. She was later started back on her anticoagulation with heparin bridging and discharged with outpatient follow-up. 

## Discussion

Endoscopic hemostasis is the gold standard and first-line treatment for bleeding peptic ulcers with a relatively low failure rate (8%-25%), followed by surgical intervention despite no significant change in the rate of mortality [3]. A bridge between endoscopic and surgical intervention is endovascular coiling or transcatheter arterial embolization (TAE). This is an effective and safe method to control the bleeds or rebleeds after the failure of conventional endoscopic interventions with low mortality in patients with coagulation dyscrasias, antiplatelet therapies, liver dysfunction, and on chronic anticoagulation [4]. The typical indications for TAE include massive bleeding (transfusion requirement ≥ four units of blood per 24 h), hemodynamic instability secondary to blood loss, failure to conservative medical therapy [volume replacement, antacids, H2 receptor blocking agents, or proton pump inhibitors (PPIs), and have failed attempts for endoscopic intervention to control the bleeding] [5]. Embolic agents in TAE can be either coil, gelatin sponge, polyvinyl alcohol (PVA), N-butyl 2-cyanoacrylate (NBCA) glue, or ethylene-vinyl alcohol copolymer [6]. The use of coils and micro coils had shown lower infarction rates, and studies had shown better results when coils were used with any of the above-mentioned embolic agents, especially PVA and gelatin sponge in patients with coagulopathies [6]. Our patient also underwent a similar procedure due to underlying coagulopathy. Coils are used in a very precise fashion and carry a low risk of infarction secondary to preserving the distal microvasculature because of their size and length ratio to the vasculature [2]. Use of NBCA embolotherapy (NBCA) and a gelatin sponge alone is safe except for increased chances of rebleeds with using just a gelatin sponge which is a cheaper option [7]. The outcome of doing TAE depends upon the etiology of the bleed. Prophylactic TAE has more chances of rebleeds than therapeutic ones [8]. Overall complications after getting a TAE range from groin hematomas to contrast-induced nephropathy (CIN). The outcome of embolization in upper gastrointestinal bleeds has been reportedly better than the lower gastrointestinal bleeds due to a better collateral supply of vessels [2].

## Conclusions

Transcatheter arterial embolization as an alternative to endoscopic intervention for rapid gastrointestinal bleeding has been previously used as a therapeutic tool. However, it is considered more invasive than endoscopy. Although endoscopic interventions can control most bleeding ulcers, angiographic embolization can be successfully performed if endoscopic methods fail. Therefore, angiographic embolization in the setting of hematochezia or arterial bleed may be considered initially to minimize the time to therapeutic intervention. Further comparative data are required to make definitive conclusions as randomized control trials are lacking in this regard. 

## References

[1] Groenen MJ, Kuipers EJ, Hansen BE, Ouwendijk RJ (2009). Incidence of duodenal ulcers and gastric ulcers in a Western population: back to where it started. Can J Gastroenterol.

[2] Loffroy R, Favelier S, Pottecher P (2015). Transcatheter arterial embolization for acute nonvariceal upper gastrointestinal bleeding: Indications, techniques and outcomes. Diagn Interv Imaging.

[3] Wang YL, Cheng YS, Liu LZ, He ZH, Ding KH (2012). Emergency transcatheter arterial embolization for patients with acute massive duodenal ulcer hemorrhage. World J Gastroenterol.

[4] Kuyumcu G, Latich I, Hardman RL, Fine GC, Oklu R, Quencer KB (2018). Gastrodoudenal embolization: indications, technical pearls, and outcomes. J Clin Med.

[5] Loffroy R, Guiu B, D'Athis P (2009). Arterial embolotherapy for endoscopically unmanageable acute gastroduodenal hemorrhage: predictors of early rebleeding. Clin Gastroenterol Hepatol.

[6] Širvinskas A, Smolskas E, Mikelis K, Brimienė V, Brimas G (2017). Transcatheter arterial embolization for upper gastrointestinal tract bleeding. Wideochir Inne Tech Maloinwazyjne.

[7] Kim PH, Tsauo J, Shin JH, Yun SC (2017). Transcatheter arterial embolization of gastrointestinal bleeding with N-butyl cyanoacrylate: a systematic review and meta-analysis of safety and efficacy. J Vasc Interv Radiol.

[8] Sildiroglu O, Muasher J, Arslan B (2014). Outcomes of patients with acute upper gastrointestinal nonvariceal hemorrhage referred to interventional radiology for potential embolotherapy. J Clin Gastroenterol.

